# Bacterial Myocarditis in a Patient with Cancer

**DOI:** 10.31662/jmaj.2020-0082

**Published:** 2021-03-02

**Authors:** Rina Yamashita, Mizuko Tanaka, Hideki Chiba, Kotaro Sugimoto

**Affiliations:** 1 Department of Basic Pathology, Fukushima Medical University School of Medicine, Fukushima, Japan

**Keywords:** bacterial myocarditis, cardiac infection, histopathology

A 69-year-old man with stage IV esophageal cancer developed a sudden onset of fever and died of cardiopulmonary arrest. The patient had cachexia but was not in the terminal phase. Significant arrhythmia or malfunction of the heart had not been observed. An autopsy indicated that the tumor did not metastasize to any critical organs but only to the adrenal glands and peripheral lymph nodes. Meanwhile, hundreds of abscesses containing numerous gram-positive diplococci (Figure 1A, B, C and D ) were observed in the heart and lung. Thus, it was concluded that the patient died of bacterial myocarditis. Although spontaneous bacterial myocarditis is considered uncommon in developed countries, many pathogens causing pneumonia often trigger bacterial myocarditis. An autopsy study demonstrated that 40% of patients with lobar pneumonia had concomitant bacterial myocarditis ^(1), (2)^. Therefore, an understanding of the association between pneumonia and myocarditis would inform clinical practice, especially in the care for compromised patients.

**Figure 1.**
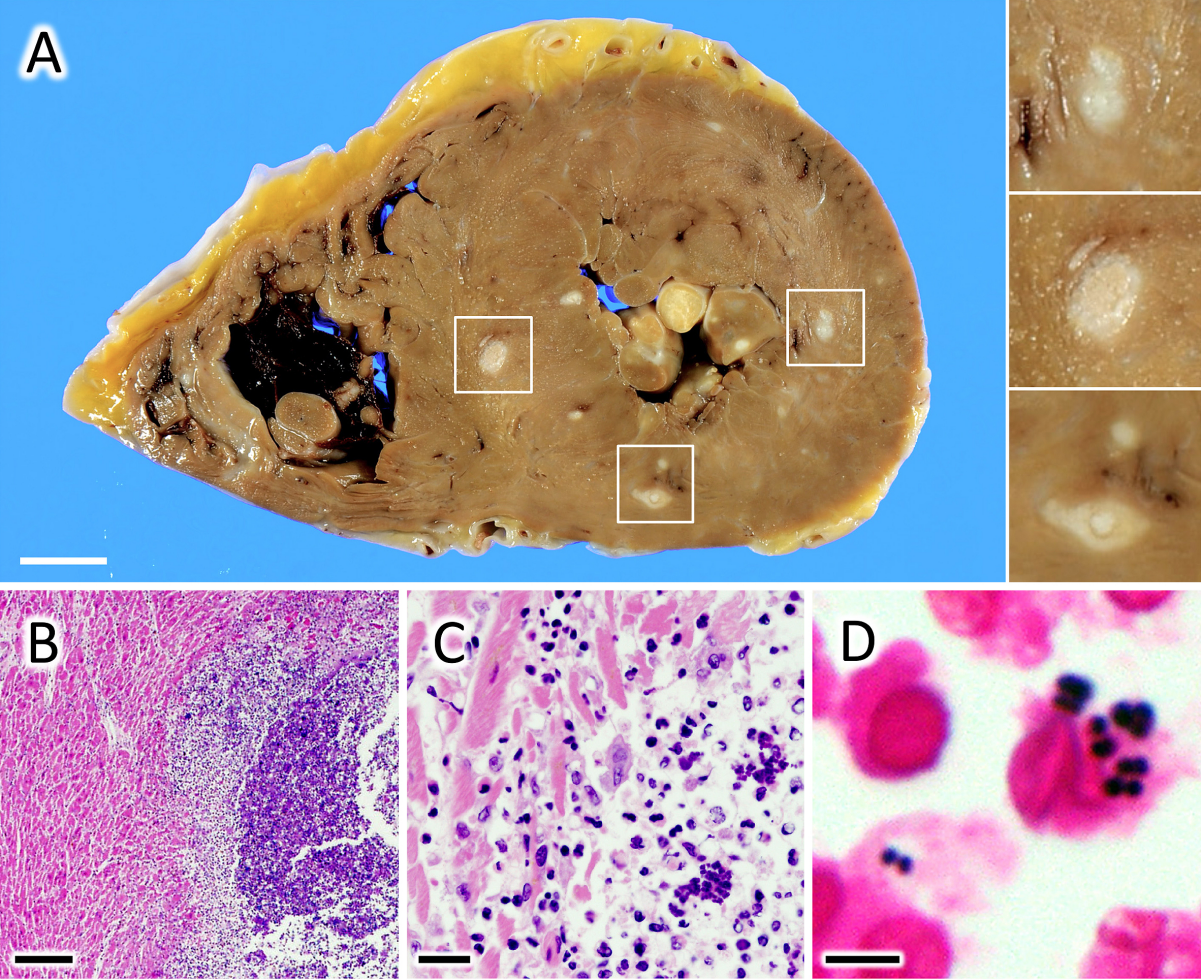
(A) Gross images of the heart. Multiple white nodules ranging in diameter from 1 to 10 mm are observed in the myocardium. Bar, 10 mm. (B-D) Microscopic images of hematoxylin and eosin (H & E; B, C) and Gram (D) staining of the white nodules. These are abscesses filled with numerous gram-positive diplococci and neutrophils. Bars, 500 μm (B), 50 μm (C), 5 μm (D).

## Article Information

### Conflicts of Interest

None

### Author Contributions

RY, MT, HC, and KS performed the autopsy, prepared the specimens, and diagnosed the patient. RY and KS wrote the manuscript.

### Informed Consent

Informed consent was obtained from the patient’s family to publish this case, including pictures.

### Approval by Institutional Review Board (IRB)

This study did not require IRB approval.
